# Through the eyes of a medical student

**DOI:** 10.1007/s40037-013-0082-z

**Published:** 2013-09-19

**Authors:** Dustin Jacobson

**Affiliations:** Michael G. DeGroote School of Medicine, McMaster University, 1280 Main St. W, Hamilton, ON L8S 4L8 Canada

**Keywords:** Art and medicine, Medical student, Patients

## Abstract

As a medical student, I have come to appreciate the generosity of the patient time that I experience. This places me in a unique position as I can become truly immersed in the perspective of the patients I see. I have the time to engage and understand how they see their illness, their social barriers and many other factors that affect their overall wellbeing. In this particular encounter, I discuss one of the more memorable interviews I’ve had with a patient. We shared a connection that I hope will influence my interactions with patients in the future.

As a medical student I am encouraged to spend ample time with patients. Aside from my learning that takes place during this time, it also gives patients the time they often need to open up emotionally. This gift of time is unique in a doctor’s career and specific to their early learning years. With patient overload, a practising doctor very often cannot afford this luxury. The connections I have made with patients because I am able to give them this amount of time, is one of the reasons I love being a medical student. I’d like to share one of these connections.

As a second-year medical student, I met Dr. M as a patient in my family medicine clinic, a part of my core rotation during my clinical clerkship. I was allotted 30 min for the interview, so that I could obtain as much information as possible. This amount of time is generous to ensure that all students are able to complete the process.

Dr. M had suffered multiple heart attacks, three over the past 8 years. He also presented with the normal risk factors: high blood pressure, high cholesterol and family history of heart disease. As well, he had painful arthritis from years of working with his hands. However, of a more urgent nature, Dr. M told me that he’s waiting for his next heart attack and, reading between the lines, I understood that he hoped that the next one would be his last, and that he would die.

Dr. M had recently emigrated from Syria where he had practised as a general surgeon. When he spoke of his time as a surgeon, he presented as a debonair man, proud of his accomplishments. He declared that he was one of the best in his field. He left Syria because of a combination of two factors. Firstly, his family was in Canada and he missed them greatly. Secondly, he was experiencing persecution in Syria. His views on medicine were apolitical and he felt compelled to help anyone who needed it. Because of this, government officials accused him of performing surgery on ‘rebels.’ This was considered traitorous. Dr. M spoke emotionally and with sincerity.

Afraid for his life, Dr. M left Syria and joined his family in Toronto. Adapting to his new life here was far from easy. While he was happy to be with his wife and children, he could not practise in Canada, he had another heart attack and English was his second language. He has tried to gain meaningful employment, but after a few job interviews he has had no luck.

My initial interview and examination of Dr. M necessitated a follow-up appointment. His blood pressure was very high and this needed to be carefully monitored. Equally concerning were his obvious signs of depression. I sat down with Dr. M again.

I could sense that Dr. M felt more comfortable with me as we had already connected the week before. I asked him to tell me more about his family.

He told me that his family is a happy one. They don’t have very much money, but they have plenty of love. He has four children, and three of the four have a rare form of neuromuscular disease. They require a lot of physical care, but are intellectually intact. They do very well in school and Dr. M is very proud of their accomplishments. He says they are on an ‘upslope’. But the fact remains, Dr. M doesn’t work, his wife doesn’t work, and he has four kids at home. He had been receiving some benefits, but navigating through a complex health care system as a newcomer is difficult. These benefits were cut and he had been without income for the past 2 months. He had just been served with an eviction notice, the first of his life and he felt helpless. He was worried for his children, worried that moving would force his children to move schools. He feared that they would be bullied by children who were not familiar with their health needs, he was afraid of bumping these children off of their ‘upslope’. He has money in Syria, but with the tremendous turmoil in the country, he could not transfer any money into Canada. He told me that 20 of his relatives had been killed since I saw him the previous week.

My task during this visit was to assess Dr. M’s depression. Using a measuring tool, we went through some of the diagnostic criteria for depression. These questions are all intensely emotional. We got to a question that talks about end of life. I asked him if he’s ever had thoughts of ending his life. He told me that he’s supposed to be too smart to do that. He didn’t say ‘no.’ He told me that life is not simply breathing. He said that life is having use. He then told me he would rather be back in Syria and working for free, with his life in danger than have no use. He stays for his family.

I asked Dr. M what made him happy in life, what stopped him from ending his life. He told me he is the provider for his family, he is responsible for them, he has many worries, he has tremendous weight on his shoulders. I asked again, ‘what makes you happy?’ He told me he enjoys when his kids tell him they love him, he enjoys their hugs, and he enjoys their kisses. At this point he broke down and cried. I too felt overcome with emotion and became tearful. Dr. M is a stoic man and was embarrassed by his reaction. I reassured him that we were talking about emotionally charged issues and these emotions are completely normal. We both reached for a tissue and we laughed together. I told him that my job is to get him onto an ‘upslope,’ similar to his daughters. He smiled.

He thanked me for the interview, thanked me for the cathartic release. I thanked him for the opportunity.

I hope that my encounter with Dr. M will always remind me to be aware that everyone has a life story. We all have hopes and dreams and for some, these don’t become a reality. I hope also to remain cognizant that simply being heard can truly impact a person who feels they live in an unkind and cold world. Every day I think about how lucky I am to be a medical student and to have the luxury of time with patients. I believe my experience with Dr. M impacted him and me in a meaningful way because of this. I didn’t do much aside from listening, but I had no other agenda. I had no patients in the waiting room. Instead, I had time; I had presence.

Through experiences like these, the utility of the medical student interview is apparent. First, from a clinical perspective, in emotionally charged situations like these, the biggest gift is time. It is rare for a patient to be willing to open up in relatively brief interactions. It is possible for a shorter interview to breech topics such as depression, but it is difficult to truly understand the severity of this illness in a ’10-minute interview.’ And, in the typical clinician’s schedule, if these topics are broached, one is not afforded the time to further delve into these questions at the time they are introduced. For example, if Dr. M’s notions of suicidality and true feelings of helplessness were not known, the close follow-up he needed may not have been put in place. Second, from an educational perspective, these early experiences shape the clinicians we will become. The skills of patience and empathy are inherent, but are still skills that need practice. In these formative years, a medical student has ample opportunity to practise these important skills, and as we transition to clinicians with busy offices, and scheduled appointments, it is important for these skills to be well tuned. In short interviews, one needs the skill to recognize and start to explore very difficult situations that take place in patients’ lives, often encompassing the psychosocial context of illness in addition to its physiological manifestations.

Dr. M is a patient that I will remember for years to come, but most importantly, the themes of patience, empathy and time will be skills that I continue to practise as I transition into the clinician I hope to become.

